# Low doses of esmolol and phenylephrine act as diuretics during intravenous anesthesia

**DOI:** 10.1186/cc11175

**Published:** 2012-01-30

**Authors:** Yu Hong Li, Hai Bin Zhu, Xiaozhu Zheng, Han Jian Chen, Liang Shao, Robert G Hahn

**Affiliations:** 1 Department of Anesthesiology, First Affiliated Hospital, College of Medicine, Zhejiang University, 79 Qingchun Road, 310003 Hangzhou, China; 2 Department of Obstetrics and Gynecology, First Affiliated Hospital, College of Medicine, Zhejiang University, 79 Qingchun Road, 310003 Hangzhou, China; 3 Zhejiang Hospital, 12 Linying Road, 310013 Hangzhou, China; 4 Yuhuan County People's Hospital, Changle Road, Yuhan County, 317600 Taizhou City, Zhejiang, China; 5 Section for Anesthesia, Linköping University, 581 85 Linköping, Sweden

## Abstract

**Introduction:**

The renal clearance of infused crystalloid fluid is very low during anaesthesia and surgery, but experiments in conscious sheep indicate that the renal fluid clearance might approach a normal rate when the adrenergic balance is modified.

**Methods:**

Sixty females (mean age, 32 years) undergoing laparoscopic gynecological surgery were randomized to control group and received only the conventional anesthetic drugs and 20 ml/kg of lactated Ringer's over 30 mins. The others were also given an infusion of 50 μg/kg/min of esmolol (beta_1_-receptor blocker) or 0.01 μg/kg/min of phenylephrine (alpha_1_-adrenergic agonist) over 3 hours. The distribution and elimination of infused fluid were studied by volume kinetic analysis based on urinary excretion and blood hemoglobin level.

**Results:**

Both drugs significantly increased urinary excretion while heart rate and arterial pressure remained largely unaffected. The urine flows during non-surgery were 43, 147, and 176 ml in the control, esmolol, and phenylephrine groups, respectively (medians, *P *< 0.03). When surgery had started the corresponding values were 34, 65 and 61 ml (*P *< 0.04). At 3 hours, averages of 9%, 20%, and 25% of the infused volume had been excreted in the three groups (*P *< 0.01). The kinetic analyses indicated that both treatments slowed down the distribution of fluid from the plasma to the interstitial fluid space, thereby preventing hypovolemia.

**Conclusions:**

Esmolol doubled and phenylephrine almost tripled urinary excretion during anesthesia-induced depression of renal fluid clearance.

## Introduction

During general anesthesia and surgery, renal clearance of crystalloid fluid is only 15% to 20% of that found in conscious volunteers [1-3]. If not carefully balanced, fluid therapy entails a risk of inducing edema. Slow elimination might be counteracted by manipulation of the adrenergic balance. In awake sheep, β_1_-receptor stimulation by isoprenaline caused fluid retention and excessive plasma volume expansion that closely resembles the kinetic situation during anesthesia and surgery. In contrast, α_1_-receptor stimulation by phenylephrine increased urinary excretion [4,5].

The aim of the present study was to examine to what degree the slow turnover of lactated Ringer's solution during anesthesia and surgery can be rectified by infusing esmolol (a β_1_-receptor blocker) or phenylephrine in patients undergoing laparoscopic gynecological surgery during intravenous anesthesia. Based on the previous animal studies [4,5], our hypothesis was that urinary excretion would increase in response to esmolol and phenylephrine. Kinetic analysis was also performed to elucidate whether the infusions cause an aberrant distribution of fluid.

Safety reasons stipulated that the amounts of drug provided be low. The rate of infusion of phenylephrine represented only 1% of that used for hemodynamic support. Esmolol was given in the starting dose recommended for the management of supraventricular tachyarrhythmias.

## Materials and methods

### Patients

Between November 2008 and October 2010, 60 American Society of Anesthesiologists (ASA) I patients between 21 and 49 (mean of 32) years of age were studied during elective laparoscopic removal of ovarian cysts or laparoscopic supracervical hysterectomy (uterus less than 1 kg) performed under intravenous general anesthesia. The protocol (reference number 080186), including the sampled blood volume of approximately 75 mL, was approved by the ethics committee of Zhejiang University (Hangzhou, China). Each patient gave her informed consent to participate.

### Procedure

Patients fasted overnight. No premedication was given. A radial artery catheter was inserted for sampling and for monitoring arterial blood pressure. Anesthesia was induced with midazolam 50 μg/kg, propofol 1.5 mg/kg, cis-atracurium 0.15 mg/kg, and fentanyl 3 μg/kg and this was followed by endotracheal intubation and mechanical ventilation. The anesthesia was maintained with propofol 6 mg/kg per hour and cis-atracurium 0.15 mg/kg per hour; fentanyl was infused at a rate of 2 μg/kg per hour during the first 30 minutes and then at 1.5 μg/kg per minute; these rates could be slightly adjusted depending on the depth of anesthesia.

An indwelling catheter was placed into the bladder. The patients were then randomly assigned by the Excel random number generator (Microsoft Corporation, Redmond, WA, USA) to receive a 3-hour continuous infusion of 10 mL/hour lactated Ringer's solution that provided no drug (controls); 50 μg/kg per minute of the β_1_-receptor blocker esmolol (Qilu Pharmaceutical Co., Shandong, China); or 0.01 μg/kg per minute of the alpha_1 _adrenergic (can we use α_1 _here instead?) receptor agonist phenylephrine (Hefeng Pharmaceutical Co., Shanghai, China). The line was primed to provide the drug from the first minute. The anesthesia and the research were managed by two anesthesiologists blinded to the study drug.

After 10 minutes for adjustment to the effects of the drug, plasma volume expansion was induced by infusing 20 mL/kg of lactated Ringer's solution (Pharmacia-Baxter, Shanghai, China) over the course of 30 minutes via an infusion pump. No other fluid (except drug vehicles) was given. No study data were collected after termination of the anesthesia.

### Measurements

Monitoring included pulse oximetry, electrocardiography, heart rate, and invasive mean arterial pressure (MAP). Data were displayed on a multifunction monitor (Datex-Ohmeda, Hoevelaken, The Netherlands) and saved digitally. The depth of anesthesia was monitored by a bispectral index (BIS) sensor applied to the forehead. The signal was recorded on a BIS monitor Model A-2000TM (Aspect Medical System, Natick, MA, USA). Urine output was measured every 20 minutes during the study. Arterial blood samples (2 mL each) were collected every 5 minutes during the first 60 minutes and every 10 minutes during the following 120 minutes. The blood hemoglobin (Hb) concentration was measured on a GEM Premier 3000 (Instrumentation Laboratory, Lexington, IL, USA). Duplicate samples collected at baseline ensured a coefficient of variation of 1.5%. Blood loss was assessed from the content of blood in suction bottles and on sponges.

### Kinetic analysis

**Figure 1:**
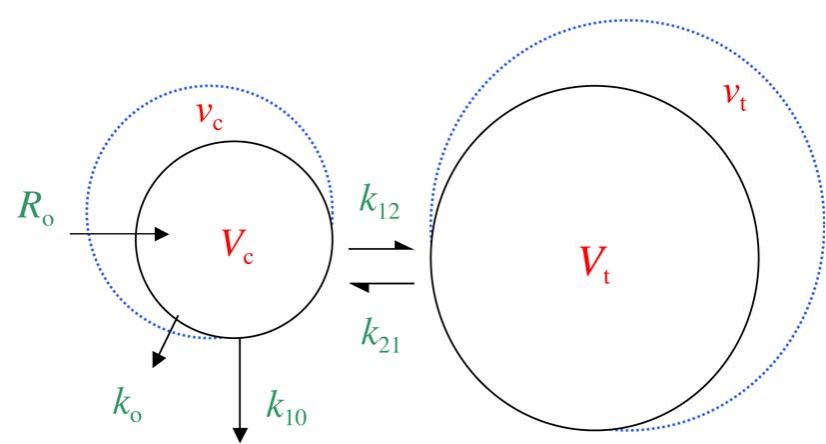
**The kinetic model used to analyze the distribution of infused fluid**. *k*_o_, fluid loss by evaporation through skin and airways; *k*_10_, rate constant for fluid leaving the system; *k*_12_, rate constant for fluid passing from *v*_c _to *v*_t_; *k*_21_, rate constant for fluid passing from *v*_t _to *v*_c_; R_o_, rate of infusion; *v*_c_, size of central body fluid space during fluid therapy; *V*_c_, size of central body fluid space at baseline; *v*_t_, size of peripheral body fluid space during fluid therapy; *V*_t_, size of peripheral body fluid space at baseline.

The distribution and elimination of fluid were analyzed by a two-volume kinetic model [1-7] with a novel adaptation that allows analysis ("analysis" is repateded word in this sentence - can we write "novel adaptation that takes uneven distribution into consideration" ?) of uneven distribution (Figure 1). Fluid was infused at rate *R*_o _to increase the volume of central body fluid space *V*_c _to *v*_c_. The rate of elimination was given as the product of the volume expansion of *V*_c _and the elimination rate constant, *k*_10 _(unit: per minute). *Perspiratio insensibilis *was accounted for by constant *k*_o_, which was preset to 0.4 mL/minute. Distribution of fluid to the peripheral body fluid space *V*_t _was governed by *k*_12 _and its return from *v*_t _to *v*_c _by rate constant *k*_21_. The differential equations are the following:

$$\begin{array}{c} \text{d}v_{c}/\text{d}t=R_{o}-k_{\text{o}}-k_{10}\left(v_{c}-V_{c}\right)-k_{12}\left(v_{c}-V_{c}\right)+k_{21}\left(v_{\text{t}}-V_{\text{t}}\right)\\ \;\;\;\;\;\;\;\;\;\;\;\;\;\;\;\;\;\;\;\;\;\;\;\;\;\text{d}v_{t}/\text{d}t=k_{12}\left(v_{c}-V_{c}\right)-k_{21}\left(v_{\text{t}}-V_{\text{t}}\right). \end{array}$$

The Hb-derived fractional plasma dilution was used to indicate the volume expansion of *V*_c _resulting from the infusion:

$$\left(v_{c}-V_{c}\right)/V_{c}=\left[\left(\text{Hb}/\text{hb}\right)-1\right]/\left(1-\text{Hct}\right),$$

where Hct stands for hematocrit. Symbols in capital letters denote baseline values. A correction for the effect of blood sampling and blood loss was made on the plasma dilution [5].

The primary parameters in the model (*V*_c_, *k*_12_, and *k*_21_) were estimated by applying a non-linear least-squares regression routine (fminsearch) by using Matlab R2010a software (The MathWorks, Inc., Natick, MA, USA), whereas *k*_10 _was calculated as a secondary parameter as follows:

$$k_{10}=\sum \text{urine}\;\text{volume}/\text{AUC}\;\text{for}\;\text{(}v_{\text{c}}\text{ - }V_{\text{c}}\text{),}$$

where AUC is the area under the curve. The renal clearance is the product of *V*_c _and *k*_10_.

### Statistical analysis

The study was powered (80%) to detect a doubling of urinary excretion in any of the study groups as compared with the controls on the basis of the ratio between the excreted urine and infused fluid previously found during surgery [2,3]. An interim analysis was made after 42 patients.

Data are presented as the median and 25th to 75th percentile range. Comparisons between the three groups were made by the Kruskal-Wallis test, which, if significant, this is so. was followed by the Mann-Whitney *U *test for *post hoc *analysis. Changes were assessed by the Wilcoxon matched-pair test. Correlations between variables were studied by simple and multiple regression analysis by using square root transformation if distribution was skewed. A *P *value of less than 0.05 was considered statistically significant.

## Results

### Demographics, hemodynamics, and anesthesia

**Figure 2:**
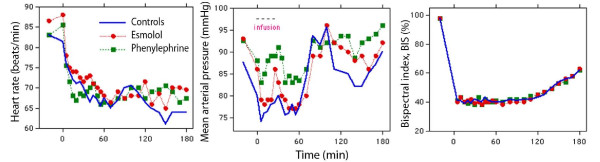
**Heart rate, mean arterial pressure, and bispectral index in patients receiving esmolol, phenylephrine, or sham (controls)**. Data are the median values.

There were no differences among the three study groups with respect to baseline parameters, operating time, blood loss (Table 1), and heart rate (Figure 2, left). Esmolol infusion was followed by a modest decrease in MAP during surgery in comparison with baseline (*P *< 0.01), whereas no change versus baseline occurred in the other two groups (Figure 2, middle). During surgery, MAP was 5 mm Hg higher in the phenylephrine group than in the esmolol group (*P *< 0.01). Phenylephrine was also associated with a slightly higher overall MAP than the other groups (Table 1). The administered amounts of anesthetic drugs were similar between the groups, but the esmolol patients received less fentanyl than the others between 60 and 120 minutes (Table 2). The BIS recordings were virtually identical (Figure 2, right).

**Table 1:** Baseline characteristics of patients and operations

Parameter	Controls (*n *= 20)	Esmolol (*n *= 20)	Phenylephrine (*n *= 20)	Kruskal- Wallis test
Age, years	32 (27-38)	32 (28-39)	33 (27-41)	NS
Body weight, kg	50 (48-55)	53 (49-59)	55 (48-60)	NS
Start of surgery, minutes	70 (65-80)	70 (70-80)	80 (60-90)	NS
End of surgery, minutes	130 (115-155)	150 (130-180)	150 (130-180)	NS
Operating time, minutes	60 (40-80)	75 (50-100)	70 (50-100)	NS
Blood loss, mL^a^	30 (30-55)	35 (25-70)	40 (30-70)	NS
MAP, mm Hg				
Baseline	88 (81-94)	92 (87-102)	92 (88-98)	NS
Non-surgery	77 (68-80)	78 (73-85)	83 (75-93)	NS
Surgery	92 (87-96)	90 (84-92)	95 (85-94)	*P *< 0.03
5 to 180 minutes	82 (59-86)	82 (80-90)	92 (85-94)	*P *< 0.02

Data are the median (25th to 75th percentile) for the group. ^a^Collected by suction and on sponges, not including blood in removed tissue. MAP, mean arterial pressure; NS, not significant.

**Table 2:** Doses of anesthetic drugs for each hour of the experiments

Measured parameters	Controls (*n *= 20)	Esmolol (*n *= 20)	Phenylephrine (*n *= 20)	Kruskal-Wallis test
Propofol, mg				
0-60 minutes	350 (279-350)	300 (300-337)	290 (250-300)	NS
60-120 minutes	316 (291-358)	324 (290-384)	320 (254-400)	NS
120-180 minutes	150 (125-225)	210 (168-249)	209 (169-271)	NS
Fentanyl, μg				
0-60 minutes	250 (250-300)	250 (213-250)	300 (250-300)	NS
60-120 minutes	200 (200-250)	150 (150-200)	216 (150-268)	*P *< 0.05
120-180 minutes	100 (100-125)	125 (100-150)	112 (100-150)	NS
Cis-atracurium, mg				
0-60 minutes	18 (15-20)	18 (15-20)	19 (15-20)	NS
60-120 minutes	9 (8-10)	10 (8-10)	9 (7-10)	NS
120-180 minutes	0 (0-0)	3 (0-5)	5 (3-5)	*P *< 0.01

Data are the median (25th to 75th percentiles). ^a^*P *= 0.11; phenylephrine versus controls *P *< 0.05 (Mann-Whitney test). NS, not significant.

### Plasma dilution

The decrease in Hb concentration during the infusions averaged 25 g/L. There were no differences in Hb-derived plasma dilution at 30, 60, or 120 minutes, but both treatment drugs were associated with a slightly less pronounced plasma dilution at 180 minutes in comparison with the controls (both differences *P *< 0.01) (Table 3).

**Table 3:** Blood hemoglobin concentration and the hematocrit

Measured parameters	Controls (*n *= 20)	Esmolol (*n *= 20)	Phenylephrine (*n *= 20)
Hematocrit at baseline, ratio	0.36 (0.35-0.39)	0.37 (0.34-0.39)	0.39 (0.37-0.31)
Hemoglobin concentration, g/L			
Baseline	112 (110-121)	113 (104-120)	121 (114-27)
30 minutes	87 (84-96)	87 (84-96)	95 (89-98)
60 minutes	96 (90-104)	95 (90-102)	102 (96-107)
120 minutes	102 (99-105)	105 (98-114)	109 (105-117)
180 minutes	102 (97-105)	105 (99-114)	112 (105-118)
Mean 5-180 minutes	98 (94-103)	99 (93-107)	105 (99-111)
Plasma dilution, no units			
30 minutes	0.45 (0.43-0.51)	0.42 (0.38-0.54)	0.45 (0.42-0.49)
60 minutes	0.30 (0.25-0.35)	0.31 (0.25-0.37)	0.30 (0.25-0.34)
120 minutes	0.18 (0.13-0.20)	0.10 (0.07-0.17)	0.12 (0.08-0.22)
180 minutes^a^	0.19 (0.15-0.25)	0.09 (0.04-0.17)	0.11 (0.03-0.17)
Mean 5-180 minutes	0.28 (0.24-0.30)	0.24 (0.20-0.29)	0.22 (0.20-0.26)

Data are the median (25th to 75th percentiles). ^a^*P *< 0.01 by the Kruskal-Wallis test.

### Urinary excretion

**Figure 3:**
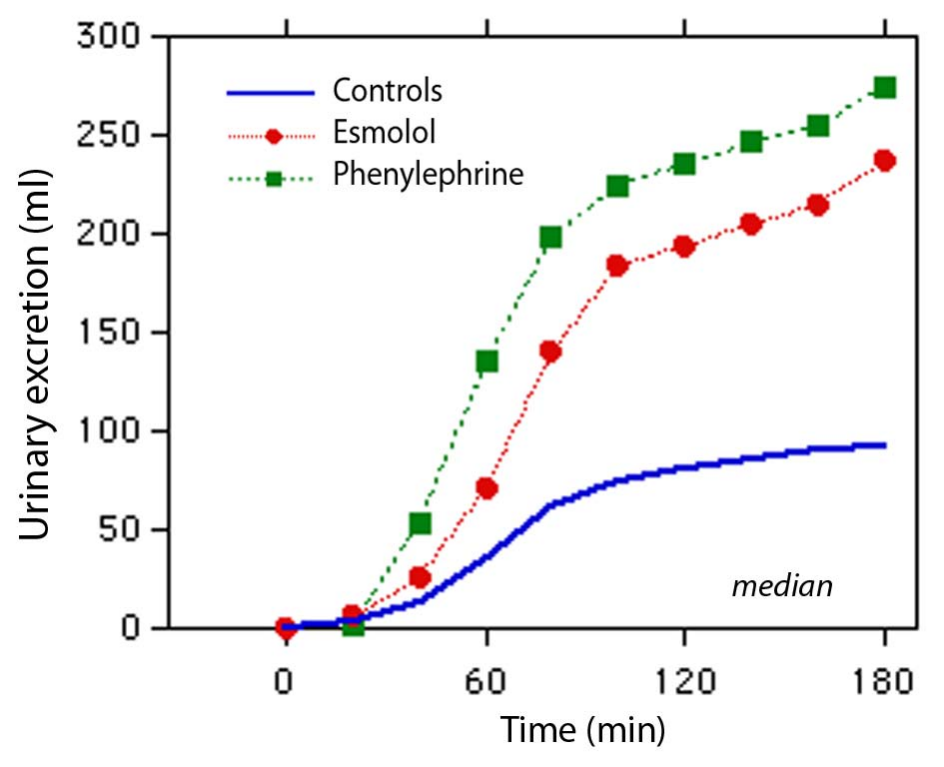
**Urinary excretion during 3 hours of anesthesia and surgery**. Twenty patients were in each group.

The cumulative urinary excretion was significantly larger in the two treatment groups than in the control group from 60 minutes onward. At 180 minutes, both treatments had more than doubled urinary excretion and the ratio between excreted and infused fluid volumes (Kruskal-Wallis test, *P *< 0.01) (Figure 3 and Table 4). Studying only the periods of non-surgery and surgery also revealed that the urinary excretion was larger and the urine flow higher in the treatment groups compared with the control group (Table 4).

**Table 4:** Detailed calculations of the urinary excretion

Measured parameters	Controls (*n *= 20)	Esmolol (*n *= 20)	Phenylephrine (*n *= 20)	Kruskal- Wallis test
Infused volume, mL	1,000 (950-1,100)	1,060 (985-1,180)	1,090 (970-1,190)	NS
Cumulative urine volume, mL				
40 minutes	13 (3-38)	26 (8-58)	54 (25-91)	NS
60 minutes	36 (15-99)	71 (43-150)	135 (58-195)	*P *< 0.05
120 minutes	81 (45-200)	194 (119-352)	236 (97-353)	*P *< 0.01
180 minutes	93 (53-226)	237 (136-416)	276 (168-375)	*P *< 0.01
Non-surgery	43 (27-142)	147 (73-228)	176 (88-285)	*P *< 0.03
Surgery	34 (24-53)	65 (46-122)	61 (39-129)	*P *< 0.04
Urine flow, mL/minute				
0-180 minutes	0.51 (0.29-1.22)	1.30 (0.76-2.31)	1.53 (0.93-2.05)	*P *< 0.01
Non-surgery	0.50 (0.25-1.18)	1.33 (0.95-2.63)	1.72 (0.86-2.70)	*P *< 0.01
Surgery	0.51 (0.33-0.83)	0.77 (0.54-1.51)	0.80 (0.54-1.34)	NS^a^
Excreted/infused at 180 minutes, percentage	8.8 (4.9-20.2)	20.4 (13.8-38.0)	25.4 (14.9-36.4)	*P *< 0.01

Data are the median (25th to 75th percentiles). ^a^*P *= 0.11; esmolol versus controls *P *< 0.05 (Mann-Whitney test). NS, not significant.

### Kinetic analysis

**Figure 4:**
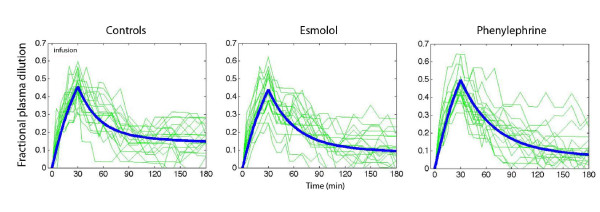
**Fractional plasma dilution during and after a 30-minute intravenous infusion of 20 mL/kg of lactated Ringer's solution in females undergoing surgery during intravenous anesthesia**. Thin lines represent one patient, and the thick line represents the modeled average. Data are based on the median kinetic data from the analysis shown in Table 5.

Figure 4 shows all individual plasma dilution-time profiles together with the optimal curve fit for each group. The three model parameters - *V*_c_, *k*_12_, and *k*_21 _- could be estimated in all experiments (Table 5). The elimination rate parameter, *k*_10_, was higher in the two treatment groups (*P *< 0.03 and *P *< 0.01) than in the control group. Moreover, *k*_21 _was lower in the phenylephrine group than in the controls (*P *< 0.02).

**Table 5:** Kinetic parameters

Estimated parameters	Controls (*n *= 20)	Esmolol (*n *= 20)	Phenylephrine (*n *= 20)	Kruskal- Wallis test
*V*_c_, L	1.5 (1.1-1.8)	1.7 (1.2-2.1)	1.6 (1.2-1.8)	NS
*k*_12_, 10^-3^/minute	28 (21-48)	22 (18-34)	18 (14-31)	NS
*k*_21_, 10^-3^/minute	11 (4-23)	7 (2-17)	4 (0-11)	*P *< 0.05
*k*_10_, 10^-3^/minute	2.1 (0.9-4.2)	4.2 (2.7-8.3)	4.7 (3.3-10.0)	*P *< 0.02

Data are the median (25th to 75th percentile) for the group during the entire experiment (0 to 180 minutes). *k*_10_, rate constant for fluid leaving the system; *k*_12_, rate constant for fluid passing from *v*_c _to *v*_t_; *k*_21_, rate constant for fluid passing from *v*_t _to *v*_c_; NS, not significant; *v*_c_, size of central body fluid space during fluid therapy; *V*_c_, size of central body fluid space at baseline; *v*_t_, size of peripheral body fluid space during fluid therapy.

**Figure 5:**
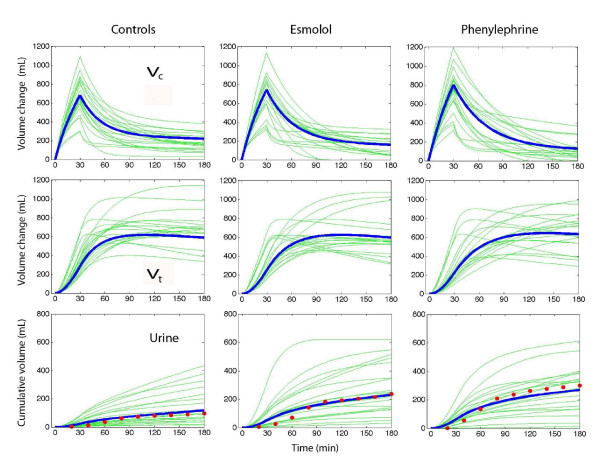
**Simulated volume-time curves for the central (*V*_c_) and peripheral (*V*_t_) fluid spaces and urinary excretion**. Thin lines are simulations based on *k*_12_, *k*_21_, and *k*_10 _from each operation, and thick lines are the median for each group. The points in the lower panel are the measured urinary excretion (median values). *k*_10_, rate constant for fluid leaving the system; *k*_12_, rate constant for fluid passing from *v*_c _to *v*_t_; *k*_21_, rate constant for fluid passing from *v*_t _to *v*_c_; *v*_c_, size of central body fluid space during fluid therapy; *V*_c_, size of central body fluid space at baseline; *v*_t_, size of peripheral body fluid space during fluid therapy; *V*_t_, size of peripheral body fluid space at baseline.

Simulations of the fluid distribution showed that the volume expansion of *v*_c _during the infusion was similar among the groups (Figure 5, top row) and virtually identical on correction for body weight. However, at 180 minutes, less fluid resided in *v*_c _in the phenylephrine group (90 mL) than in the control and esmolol groups (*P *< 0.03).

Fluid was distributed more slowly to *v*_t _in the two treatment groups. At 60 minutes, 59% of the fluid resided in *v*_t _in the control group, 48% in the esmolol group, and 43% in the phenylephrine group (*P *< 0.01; controls versus phenylephrine *P *< 0.004) (Figure 5, middle row). At 180 minutes, the volume in *v*_t _did not differ significantly. The kinetic model slightly overestimated urinary excretion early in the operations (Figure 5, bottom row).

### Linear regression analysis

Simple regression showed that MAP explained 11% of the variability in the urinary excretion (*P *< 0.02). However, multiple regression of the effects of MAP and the two treatments in turn showed that only the treatment drugs served as statistically significant predictors of the urinary excretion and the ratio of excreted/infused fluid.

## Discussion

Shifting the catecholamine balance by small amounts of drug promoted urinary excretion. In the present study, esmolol doubled and phenylephrine almost tripled the excretion of infused lactated Ringer's during laparoscopic surgery. Despite the diuretic effect, the modification of the adrenergic stimulus pattern was not strong enough to clearly affect MAP or heart rate.

The average ratio of excreted/infused fluid after 3 hours in the control group (9%) was similar to that found for acetated Ringer's during laparoscopic cholecystectomy [2] and thyroid surgery [3]. However, even with the two treatment drugs, urinary excretion was still far below the 50% to 70% of the infused volume seen within 3 hours in unstressed volunteers [6] and patients awaiting laparoscopic surgery [7]. Hence, the administered esmolol and phenylephrine were not sufficient to reverse the strong antidiuretic effect of general anesthesia and surgery. On the other hand, the present results open up for the use of these drugs when both adrenergic and diuretic effects are desired. How long the difference in urinary excretion during surgery prevails is unknown. The rate is likely to increase slightly when anesthesia is terminated [2], but much of the fluid retained during surgery remains in the body for several days [8]. In contrast, infusions of crystalloid salt [7] and glucose [9] solution initiated several hours later are excreted effectively.

Why the diuretic response to plasma volume expansion is blunted during anesthesia and surgery is poorly understood but probably can be attributed to vasodilatation, reduced arterial pressure, and activation of the renin-aldosterone hormonal axis [1]. Beta-receptor activity may also play a part. The renal clearance of crystalloid fluid is slightly higher during open laparotomy [10] than during laparoscopy [2]; therefore, body position and pneumoperitoneum might also depress the renal excretion of fluid.

The physiological effects of intravenous anesthesia also promote a pronounced plasma volume expansion in response to crystalloid fluid. When the infusions ended, 60% to 70% of the infused fluid remained in the functional plasma volume, *v*_c_. Similarly high percentages have been reported during isoflurane anesthesia [11] and during general anesthesia and surgery [2,3,10]. Pronounced plasma volume expansion during infusion implies that *V*_c _is low, and this is typical of anesthesia-induced vasodilatation [1]. Here, the small *V*_c _can also be explained by the relatively low body weight (50 to 55 kg) of the Chinese women studied. The study drugs might have altered *V*_c_, although the average volume in the esmolol and phenylephrine groups differed very little from that of the controls.

A key finding of the kinetic analysis is that both study drugs slowed down the distribution of fluid from *v*_c _to *v*_t _and this probably reflects minimally decreased filtration due to vasoconstriction. This counteracted any hypovolemic effect of the increased diuresis during the first half of the experiments. The fluid volumes residing in *v*_c _became reduced at the end of the study, but, at that time, differences were hardly worth noting. The fluid volumes in *v*_t _did not differ significantly at 180 minutes (Figure 5).

Variable results for the diuretic effect of esmolol in previous work suggest that the present study may be relevant only to anesthetized subjects, possibly because the renin-aldosterone axis is then stimulated. Complete β_1_-receptor blockade in conscious humans lowers the resting plasma levels of renin, angiotensin, and aldosterone but without affecting the diuretic response to volume loading with saline [12]. In awake sheep, esmolol reduced the diuretic response to infused fluid [13]. Moreover, more fluid tended to accumulate in the extravascular space [13] and this was not encountered during intravenous anesthesia in humans (Figure 5).

Phenylephrine increases the urinary excretion by pressure diuresis if the increase in MAP is profound, but also by increasing the release of atrial natriuretic peptides [4]. The small doses of drugs used in the present study make the role of these mechanisms speculative. These doses are, in fact, also a limitation since we cannot outline the diuretic effects of larger amounts. In sheep, larger doses of phenylephrine markedly lower the plasma volume [4,5] but this was not an issue in the present study (Figures 4 and 5).

Another limitation was that the operations were performed on ASA I patients only. Therefore, the effects of the study drugs on patients with cardiovascular disease are unclear. Operations were also performed in a teaching hospital and this explains why more than 1 hour had passed before the surgery started. The restoration of baseline MAP between 70 and 90 minutes in all groups can be understood on the basis of the late start of the surgery (Figure 2, middle). Hence, the entire infusion and the 30-minute post-infusion period of distribution usually took place before surgery began. However, plasma volume expansion did not change abruptly when surgery started; this is not the case during emergence from anesthesia for laparoscopy [2,14].

The main conclusion still appears to be valid when the urinary excretion is calculated for periods of apparently different MAPs and for periods of surgery and non-surgery (Tables 2 and 4). The greatest difference in urinary excretion occurred early during the procedures (that is, during periods of non-surgery, when the plasma dilution was most pronounced). The excreted volumes were smaller when surgery eventually started (and MAP was higher). Some statistical differences in urinary excretion were lost during the second half of the study. However, the average values for the groups still showed a continued trend from the first half of the experiments, namely that the treatment drugs were associated with a larger urinary excretion.

The novel kinetic analysis used here has the advantage of being able to estimate all parameters without having to discriminate between two models [1]. Drobin's model was first fitted to the data [15] but did not yield reasonable estimates for all curves. A drawback of the present approach is that the urinary excretion needs to be measured. Model stability is also increased by the fact that the size of *V*_t _is not estimated, although it can be inferred in retrospect. The output is given in the form of *V*_c _and three rate constants. The latter the three rate constants are implied here should be multiplied by *V*_c _to be converted into clearances, which is used in virtually all previous publications of volume kinetics. Yes, it should be "are". I originally meant clearance as a concept, but I guess we should aim at them being three different types of clearance.

## Conclusions

Low doses of esmolol doubled and phenylephrine almost tripled urinary excretion. These drugs may potentially be used as diuretics when renal fluid clearance is reduced by anesthesia and surgery-associated stress. Further work is needed to elucidate how the diuretic effects of these drugs are best employed. Phenylephrine might be an alternative to a conventional diuretic such as furosemide if the hemodynamics is unstable. Esmolol could be used as a mild diuretic in patients with supraventricular arrhythmias.

## Key messages

• The clearance of crystalloid fluid is very low during intravenous anesthesia.

• Small doses of esmolol and phenylephrine increase the clearance but not up to the normal values for conscious volunteers.

• These drugs at least doubled the urinary excretion in comparison with controls.

• The increased diuresis did not cause hypovolemia as both esmolol and phenylephrine slowed down the rate of distribution of lactated Ringer's from plasma to interstitium.

## Abbreviations

Hb: hemoglobin; MAP: mean arterial pressure; *k*_o_: fluid loss by evaporation through skin and airways; *k*_10_: rate constant for fluid leaving the system; *k*_12_: rate constant for fluid passing from *v*_c _to *v*_t_; *k*_21_: rate constant for fluid passing from *v*_t _to *v*_c_; R_o_: rate of infusion; *v*_c_: size of central body fluid space during fluid therapy; *V*_c_: size of central body fluid space at baseline; *v*_t_: size of peripheral body fluid space during fluid therapy; *V*_t_: size of peripheral body fluid space at baseline.

## Competing interests

The authors declare that they have no competing interests.

## Authors' contributions

YHL wrote the ethics application, organized the study, and collected data. HBZ, XZ, HJC, and LS collected data and performed the operations. RGH designed the trial, performed the calculations, and wrote the manuscript. All authors read and approved the final manuscript.
